# Soft Food

**Published:** 1875-02

**Authors:** 


Soft Food.—It is folly to put soft food into a mouth provided with good teeth. It is bad enough to allow the cook to do our mastication for us when we are old and feeble and toothless, but it is wicked for us to spare ourselves that labor so long as we are able to do it for ourselves. We once knew a good, motherly soul—and we are sure there are millions of them—who always fixed up a nice, tempting plate of milk toast when any of the family were sick. It was pleasant to the palate, abounded in butter, and was always acceptable, even when the appetite was delicate. The good lady was wrong. A slice of bread, which had been baked two or three days before, toasted very brown but not burned, with no butter, would have been a thousand times better for the patient, and would, in fact, be a thousand times better for anybody, sick or well. No article of food will bring on a sick-headache more expeditiously than milk toast. Nearly all soft food will do this with a good deal of promptness, but milk-toast is the prize substance for the purpose. Whenever we are able to fairly inculcate the principle that digestion naturally begins in the mouth, instead of the stomach, we shall do an enormous work in the way of saving human life. Soft food secures no admixture with the needed saliva, and therefore reaches the stomach quite unprepared for entrance there. It is safe to say that any human being can live healthily on half the amount of food, well masticated, which would be required if "bolted" into the stomach in the common way. The reason is, that the whole of the well-masticated mass would go to make chyme and chyle and muscle, while much of the latter would be voided unused.

---

A Punctured Brain.—Many months ago a lad, 16 years old, was accidentally wounded by the discharge of a pistol in the hands of a companion a few feet distant. Upon receiving the shot, the boy fell with violence, but did not lose consciousness. The ball, about the size of a buckshot, entered the right frontal bone an inch above the center of the eye-brow, and, passing through the brain, lodged in the occipital bone near the center of the occipital cross. A silver probe passed by its own weight to the center of the brain, without touching the ball. The wound healed rapidly, without any constitutional disturbance, and in ten days he was out. He is in good health, and has never suffered the slightest inconvenience from the accident.

---

Coffee starch is an excellent starch for black calicoes and colored linens, much better than that made with water, for it increases rather than lessens the depth of the color. Take a cup of strong coffee, boiling hot, and turn it upon two tablespoonsful of starch mixed with just enough water to make it into a thin, smooth paste. Let it boil for 15 or 20 minutes, and stir it around two or three times with a paraffine or spermaciti candle. When nearly cold starch dark colored calicoes, black muslins and brown linens with it.

